# Biopolymer-Based Gel Capsules for Improved Probiotic Delivery

**DOI:** 10.3390/gels12030212

**Published:** 2026-03-04

**Authors:** Roxana Elena Gheorghita, Andrei Lobiuc, Mihai Covasa, Alina Crina Muresan, Ioan Ovidiu Sirbu

**Affiliations:** 1 Faculty of Medicine and Biological Sciences, Victor Babes University of Medicine and Pharmacy Timisoara, 300041 Timisoara, Romania; roxana.puscaselu@usm.ro (R.E.G.); ovidiu.sirbu@umft.ro (I.O.S.); 2 Department of Biological and Morphofunctional Sciences, College of Medicine and Biological Science, Stefan cel Mare University of Suceava, 720229 Suceava, Romania; andrei.lobiuc@usm.ro; 3 Department of Foundational and Clinical Sciences, Thomas F. Frist Jr. College of Medicine, Belmont University, Nashville, TN 37212, USA; 4 Interdisciplinary Research Centre, Eco-Nano Technology and Advance Materials CC-ITI, “Dunarea de Jos” University of Galati, 800008 Galati, Romania; 5 Faculty of Engineering, “Dunarea de Jos” University of Galati, 800008 Galati, Romania; 6 Center for Complex Network Science, Victor Babes University of Medicine and Pharmacy Timisoara, 300041 Timisoara, Romania

**Keywords:** sodium alginate, starch, *Lacticaseibacillus rhamnosus*, *Bacillus clausii*, encapsulation

## Abstract

Biopolymer-based encapsulation represents an effective strategy to enhance probiotic stability and targeted gastrointestinal delivery. In this study, gel capsules composed of sodium alginate (SA) and wheat starch (ST) were developed via extrusion to encapsulate *Lacticaseibacillus rhamnosus* (*L. rhamnosus*) and *Bacillus clausii* (*B. clausii*), aiming to improve probiotic viability and controlled release. Capsule morphology, color, swelling behavior, encapsulation efficiency, and probiotic survival under simulated gastrointestinal conditions were systematically evaluated as a function of polymer ratio and probiotic loading. Capsule diameters ranged from 236.6 to 279.17 μm and were primarily governed by the SA-ST ratio, with higher ST content yielding smaller, more compact structures. Encapsulation efficiency varied between 71.2% and 96.7%, reaching maximal values in formulations with balanced SA:ST ratios (1:1) and higher probiotic loads. All formulations maintained high cell viability (>96%) following encapsulation. In vitro digestion studies demonstrated that SA-ST capsules significantly enhanced probiotic survival in simulated gastric and intestinal fluids, with the highest cumulative survival observed in ST-rich matrices containing 20% probiotic load. Swelling analyses revealed that ST incorporation promoted controlled hydration and matrix relaxation without compromising structural integrity, supporting sustained release behavior. Overall, the SA-ST biopolymer system provides a simple, scalable, and cost-effective platform for co-encapsulation of *L. rhamnosus* and *B. clausii*, offering synergistic protection, high encapsulation efficiency, and improved gastrointestinal stability, with promising applications in functional foods and pharmaceutical formulations.

## 1. Introduction

Encapsulation of probiotic bacteria is increasingly employed to enhance their stability and viability in acidic food matrices, such as yogurt. In this approach, the active materials (bacterial cells) are entrapped or embedded within a coating material that forms a protective barrier. This barrier isolates the microorganisms from environmental stressors, including acidity, oxygen exposure, and gastrointestinal conditions, thereby reducing cellular damage. Moreover, encapsulation enables targeted release in the gastrointestinal tract, helping to maintain the stability and viability of probiotic cultures during processing, storage, and digestion [1].

Currently, the main techniques used for probiotic encapsulation include extrusion, emulsion, and spray drying. Among these, extrusion is widely preferred due to its low cost, mild processing conditions, and avoidance of high temperatures or organic solvents. In addition, the materials employed in extrusion-based systems are generally recognized as safe. The capsule walls are typically composed of food-grade polymers that protect the encapsulated contents from external environments. Suitable polymers for microencapsulation by extrusion include SA, chitosan, milk proteins, and various polysaccharide gums. Among these, SA—a natural, anionic, and hydrophilic biopolymer—is the most commonly used owing to its excellent biocompatibility, biodegradability, and low cost [2,3].

Previous studies have demonstrated that ST nanoparticles are not digested in the small intestine but are metabolized in the colon, where they exert prebiotic effects [4]. As a result, ST-based materials have attracted increasing interest in the food, pharmaceutical, and agricultural industries due to their biocompatibility, wide availability from natural sources, and ease of chemical or physical modification [5]. ST and its derivatives are particularly appealing as encapsulating agents because of their ability to incorporate a broad range of bioactive compounds, while simultaneously providing nutritional benefits and improving consumer acceptability. Consequently, ST-based systems may help overcome common challenges such as loss of bioactivity, reduced absorption, or degradation of active substances under environmental and physiological conditions [6].

Probiotics are increasingly studied for their health benefits and are defined as live microorganisms that, when administered in adequate amounts, confer benefits to the host. Growing evidence supports their role in the management of gastrointestinal disorders, liver disease, obesity, diabetes, cancer, and other conditions [7]. However, many probiotic strains remain highly sensitive to gastrointestinal stress, highlighting the need for effective delivery systems that preserve viability and functionality.

*Lacticaseibacillus rhamnosus* (*L. rhamnosus*) is a well-established probiotic known for its role in maintaining intestinal health and microbial balance. This Gram-positive, facultative anaerobe colonizes the small intestine, tolerates gastric and biliary conditions, and promotes mucosal adhesion through extracellular polysaccharides and surface fimbriae [8]. Clinically, *L. rhamnosus* has been associated with improved gastrointestinal function, immune regulation, metabolic health, and protection against liver and inflammatory disorders, and is widely used in dietary supplements and infant formulae [8,9,10,11].

*Bacillus clausii* (*B. clausii*) is a Gram-positive, spore-forming probiotic characterized by exceptional resistance to acidity, bile, heat, and processing conditions. Its spores ensure survival during gastrointestinal transit, while its non-transferable antibiotic resistance profile supports safe clinical use [12]. *B. clausii* contributes to intestinal barrier integrity, immune homeostasis, and pathogen inhibition through the production of antimicrobial peptides such as claucin [12,13].

Building on previous work in probiotic encapsulation, this study proposes an original biopolymer-based system using sodium alginate (SA) and starch (ST) to co-encapsulate *L. rhamnosus* and *B. clausii*. To our knowledge, this specific formulation has not been previously reported. SA provides structural stability and pH-responsive release, while ST offers a cost-effective, hydrophilic component that enhances matrix functionality [14,15]. Compared with systems based on chitosan, carrageenan, or protein- and lipid-based matrices, the SA-ST combination offers advantages in cost, availability, processability, and probiotic viability preservation [16].

The novelty of this work also lies in the synergistic co-encapsulation of *B. clausii* and *L. rhamnosus*. *B. clausii* provides rapid antimicrobial and microbiota-modulating effects, while *L. rhamnosus* supports sustained colonization and immunomodulation. Encapsulation enables their simultaneous intestinal delivery, potentially enhancing probiotic efficacy through complementary and sequential actions. Overall, the proposed encapsulation strategy is simple, reproducible, and scalable, enabling the production of stable, biocompatible, and controlled-release probiotic formulations. This approach combines technological efficiency with economic feasibility and supports the development of functional food and pharmaceutical products with improved probiotic stability and biological activity.

## 2. Results and Discussion

### 2.1. Physical and Optical Characterization of Capsules

The mean capsule diameters ranged from 236.59 ± 0.24 μm to 279.17 ± 1.12 μm. Probiotic incorporation significantly influenced capsule size across all formulations. Specifically, sample 1 (SA:ST = 2500:500, without probiotics) exhibited the largest mean diameter (279.17 ± 1.12 μm), whereas sample 9 (SA:ST = 1500:1500, 20% probiotics) showed the smallest diameter (236.59 ± 0.24 μm). Intermediate values were observed for samples 4 (2000:1000, control) and 7 (1500:1500, control), confirming the progressive size reduction with decreasing SA content and increasing probiotic load.

Increasing ST content, accompanied by reduced SA levels, resulted in progressively smaller capsule diameters (sample 1 > sample 4 > sample 7). Consistently, the smallest capsules were obtained in formulations combining lower SA content with higher ST and probiotic loads, with samples 8 and 9 showing the lowest diameters (242.32 ± 0.31 μm and 236.59 ± 0.24 μm), significantly different from formulations with higher SA content. The low standard deviations across all samples indicate good reproducibility and minimal measurement variability.

**Figure 1:**
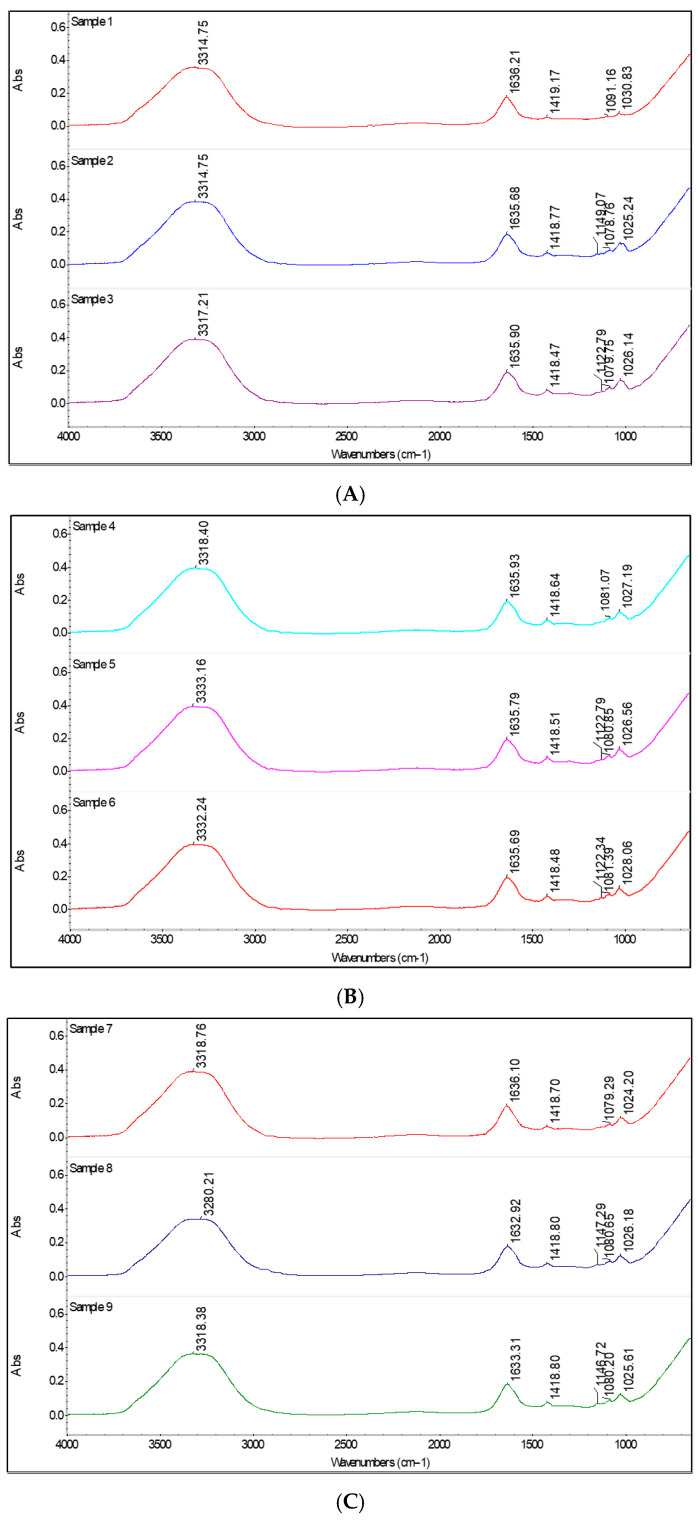
FTIR spectra of capsules: (**A**) control (Samples 1, 4, and 7), (**B**) 10% probiotic (Samples 2, 5, and 8), and (**C**) 20% probiotic (Samples 3, 6, and 9).

FTIR spectroscopy was used to examine interactions between the probiotics and the biopolymeric matrix in the probiotic-loaded microbeads. Infrared absorption spectra were recorded to characterize and identify the constituent materials. This technique exploits the characteristic absorption of infrared radiation by specific molecular groups within the 1000–4000 cm^−1^ range. The FTIR spectra confirmed the presence of the expected functional groups and indicated chemical interactions between the probiotics and the encapsulating matrix (Figure 1).

As shown in Figure 1A, the three spectra display similar profiles, indicating that formulations with a high sodium alginate content are structurally stable and that probiotic incorporation does not disrupt the polymer network. Minor differences in O–H stretching intensity (~3300 cm^−1^) are observed, while the polysaccharide region (1100–1000 cm^−1^) increases slightly with higher probiotic content. The carboxylate bands (~1600–1400 cm^−1^) remain well defined across all samples, confirming high structural integrity.

In samples 4–6 (Figure 1B), spectral differences become more pronounced. Higher starch content increases matrix hydration, an effect further enhanced by probiotic addition, as evidenced by the marked increase in O–H intensity in the sample containing 20% probiotics (sample 6). The most pronounced differences are observed in Figure 1C. The control sample (sample 7) exhibits low O–H intensity and well-defined carboxylate bands, whereas increasing ST content enhances hydration, resulting in a softer, more swollen network more susceptible to rapid intestinal release.

In all probiotic-loaded samples, a new band at 1120–1140 cm^−1^ appears that is absent in control samples, corresponding to C–O–C and C–O–H vibrations. This band suggests chemical or physical interactions between the polysaccharide matrix and probiotic cell components, such as exopolysaccharides or surface proteins. Similar findings were reported by Shoukat et al. in starch-based capsules containing *L. rhamnosus*, where peaks near 1140 cm^−1^ were attributed to peptidoglycans, a major component of Gram-positive bacterial cell walls [7].

The broad absorption band around 3300 cm^−1^, present in all samples, corresponds to O–H stretching vibrations from ST and SA. Its persistence in both control and probiotic-loaded capsules indicates preservation of the polysaccharide matrix upon probiotic incorporation and confirms the suitability of all formulations for encapsulating biologically active compounds. Comparable observations were reported by Jafari et al., who associated bands in the 3100–4000 cm^−1^ range with O–H and N–H stretching vibrations and bands between 1700 and 1300 cm^−1^ with lipid and protein components [17].

**Figure 2:**
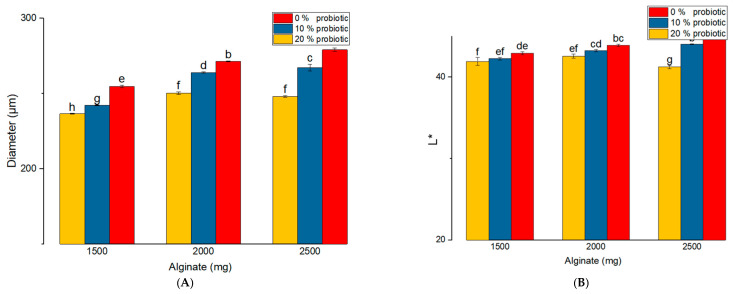

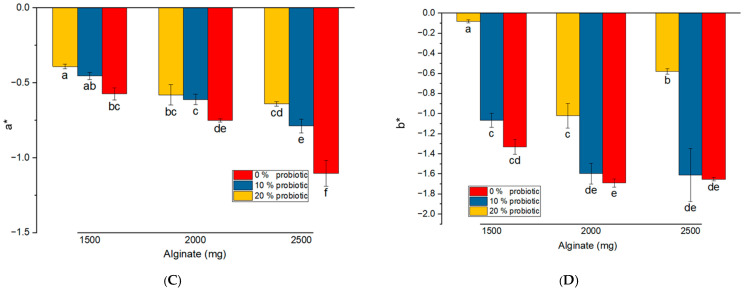
Influence of formulation variables on capsule size and color parameters. (**A**) Mean capsule diameter (μm); (**B**) luminosity (L*) values; (**C**) a* values (green–red axis of CIELab); and (**D**) b* values (blue–yellow axis of CIELab). Data are presented as mean ± SEM. Different letters indicate statistically significant differences (*p* < 0.05).

**Figure 3:**
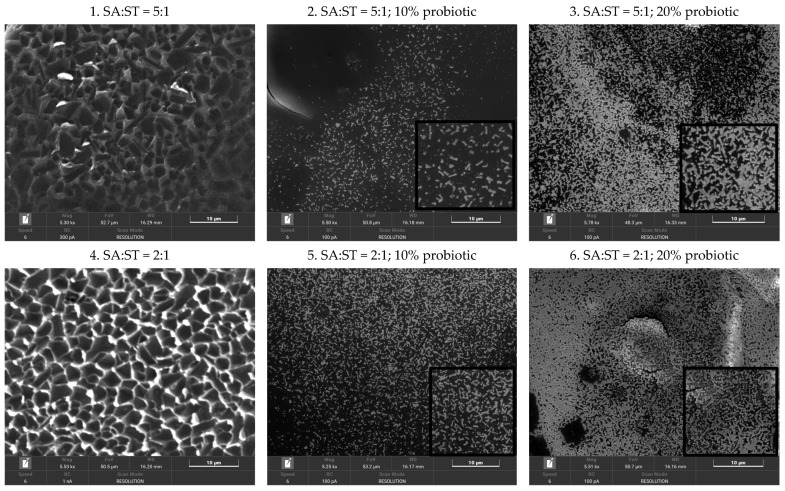

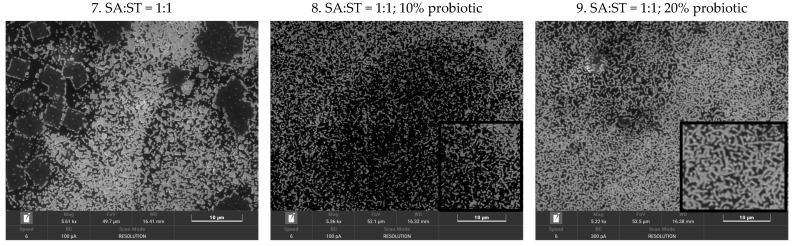
Representative scanning electron microscopy (SEM) micrographs of control and probiotic-loaded capsules. In control samples (1, 4, and 7), the capsule morphology is clearly visible, whereas bacilli are evident in capsules containing 10% probiotics (sample 2, 5, and 8) and 20% probiotics (sample 3, 6, and 9). Insets highlight the presence of bacilli at higher magnification. ST, starch; SA, sodium alginate.

As shown in Figure 2A, capsule diameter was primarily influenced by the SA-ST ratio and probiotic load (*p* < 0.001). Higher SA concentrations (2500–2000 mg) combined with lower ST content produced larger capsules, whereas decreasing SA and increasing ST resulted in progressively smaller particles. For blank formulations, the mean diameter decreased from approximately 279 μm at 2500 mg SA (sample 1) to 254 μm at 1500 mg SA (sample 7), indicating that ST incorporation promotes the formation of a more compact matrix. Increasing the probiotic load to 20% further reduced capsule size compared with the 10% formulations (263.97 μm vs. 248.06 μm for probiotic samples with SA:ST in 5:1 ratio, 263.33 μm vs. 253.43 μm for probiotic samples with SA:ST = 2:1, and 270.32 μm vs. 250.13 for equal amount of biopolymers into composition). This is likely due to bacterial cells occupying free volume within the polymer network and limiting matrix expansion during gelation (Figure 3).

Microscopy images reveal clear differences in capsule structural organization as a function of the SA-ST ratio (Figure 3). Capsules with a 5:1 SA-ST ratio (sample 1) exhibit a less dense and poorly organized matrix with non-uniform regions. At a 2:1 ratio (sample 4), the matrix becomes more compact and homogeneous, indicating improved biopolymer interactions and enhanced structural stability. Capsules with a 1:1 SA-ST ratio (sample 7) display a dense, well-organized network with high compaction and uniform phase distribution, highlighting the key role of starch in densifying the polymer matrix. Increasing starch content promotes network compaction, primarily due to the abundance of hydroxyl (–OH) groups in starch, which form hydrogen bonds with alginate carboxylate groups. In contrast, ionically cross-linked alginate (via Ca^2+^ from CaCl_2_) tends to form a more flexible and open network. Thus, higher starch proportions favor the formation of a continuous phase, resulting in a more compact and structurally stable matrix.

SEM analysis confirmed the presence and distribution of probiotics within the capsule matrix (Figure 3). The micrographs clearly confirm the presence of probiotics in capsules containing 10% and 20% loadings. Owing to their protein-rich structure and functional groups (–NH, –COO^−^; Figure 1), probiotics occupy free spaces within the polymer network, limiting polymer chain reorganization. Interactions between bacterial cell walls and polymer chains further restrict chain mobility, resulting in increased matrix densification. Consequently, probiotic-loaded samples exhibit progressively denser matrices with increasing starch content (sample 8 > sample 5 > sample 2), while higher probiotic loading (20%) leads to near-complete occupation of the polymer network (sample 9 > sample 6 > sample 3). The resulting compact structure enhances resistance to gastric and enzymatic degradation and supports probiotic survival under low-pH conditions, while reducing structural defects. Similar results were reported by Pirarat et al. for *L. rhamnosus* encapsulated in alginate-based beads [18]. Importantly, probiotics in the present study retained their cellular morphology, in contrast to spray-dried systems where structural deformation has been reported [19].

Overall, polymer composition emerged as the primary determinant of capsule size, a parameter known to influence both encapsulation efficiency and release kinetics. Comparable trends were reported by Oberoi et al., who examined the effect of SA combined with various encapsulating agents on the survival of *L. rhamnosus*, observing the smallest capsules with acacia gum (188.2 ± 4.98 μm) and the largest with sodium caseinate (~1370.3 ± 126.7 μm) [20]. Similarly, Mirtic et al. reported increased capsule diameters with higher SA concentrations, although they attributed relatively small size differences, comparable to those observed here (maximum ~24 μm), primarily to processing parameters, particularly nozzle diameter during extrusion [21].

Large particle size may reduce the survival of microencapsulated bacteria by increasing pore size within the hydrogel matrix, facilitating the diffusion of oxygen, acids, bile salts, and digestive enzymes that can inactivate cells. Although larger beads can provide enhanced protection against environmental stressors, excessively large capsules may adversely affect texture and consumer acceptability. Capsule size is therefore a critical determinant of release behavior: smaller capsules, owing to their higher surface-to-volume ratio, typically release their contents more rapidly, whereas larger capsules exhibit prolonged gastric residence and slower release. For instance, beads approximately 3.5 mm in diameter rapidly transit from the stomach to the intestine, whereas larger capsules (~12 mm) are retained longer and release their contents only after an increase in bolus viscosity [22].

In the present study, capsule diameters did not exceed 279.17 µm, minimizing gastric retention and supporting their suitability for targeted and sustained intestinal delivery. Formulations with higher SA-ST ratios produced slightly larger beads, which may modulate release kinetics, whereas capsules with balanced polymer ratios yielded smaller particles likely to provide more uniform protection and controlled release. In addition, Sa-rich capsules are more susceptible to gastric degradation, while the use of medium-viscosity SA favors the formation of porous structures that enhance fluid uptake and controlled dissolution.

Color analysis revealed that both matrix composition and probiotic addition significantly influenced capsule appearance. Luminosity (L*) values ranged from 41.25 ± 0.22 for sample 3 (20% probiotic) to 44.95 ± 0.14 for the control sample with the highest SA content (Sample 1). Probiotic incorporation exerted a more pronounced effect in formulations with higher SA levels, as evidenced by the largest luminosity variation (3.7 units) observed among samples 1–3. In general, the addition of probiotics resulted in lower L* values, indicating darker capsules. For formulations containing 10% probiotics, luminosity progressively decreased with increasing ST content (sample 2: 44.06 ± 0.05 > sample 5: 43.25 ± 0.12 > sample 8: 42.27 ± 0.16), a trend that was also observed in samples with 20% probiotic loading. As shown in Figure 2B, capsule luminosity differed significantly among formulations, with a significant interaction between polymer composition and probiotic concentration (*p* < 0.001). Capsules with higher SA content were lighter in color, whereas increased ST content or higher probiotic loading resulted in darker shades. These findings underscore the strong influence of formulation composition on capsule appearance and are consistent with previous reports showing that SA-ST capsules exhibit lower luminosity compared with SA-only systems [23].

The a* values, representing the green–red axis of the CIELab color space, differed significantly among formulations (Figure 2C). Sample 1 exhibited the most negative a* value (−1.10 ± 0.08), corresponding to a slightly greener hue. In contrast, formulations containing 10% probiotics (samples 2, 4, 6, and 8) showed progressively less negative values (−0.79 ± 0.04; −0.75 ± 0.005; −0.58 ± 0.06; and −0.45 ± 0.02), indicating a shift toward red. The highest a* values were observed in ST-rich formulations (samples 7–9), with sample 9 (−0.39 ± 0.01) displaying the reddest hue. Overall, higher SA content was associated with greener coloration, whereas increased ST content and probiotic loading shifted capsule color toward red. For the blue–yellow axis (b*), all values were negative, indicating a bluish tint (Figure 2D). Probiotic concentration was the dominant factor influencing b* values (F = 73.04), with increasing probiotic load (20%) producing a significant shift toward yellow (less negative values). Sample 4 exhibited the most pronounced blue hue (−1.69 ± 0.04), whereas sample 9 showed the highest b* value (−0.08 ± 0.01), corresponding to a more yellowish appearance. No significant interaction between polymer composition and probiotic load was observed for this parameter (*p* > 0.05).

**Figure 4:**
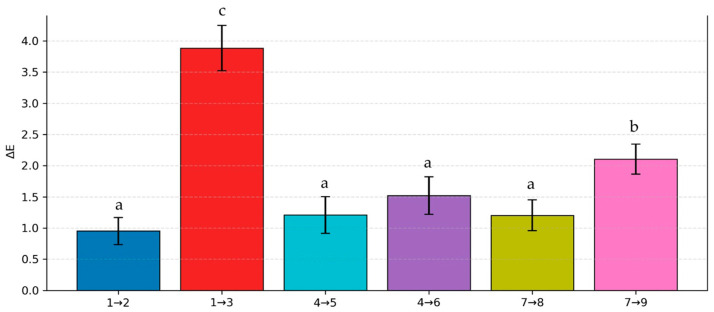
Color differences (ΔE) between control formulations (samples 1, 4, and 7) and probiotic-loaded capsules (samples 2, 3, 5, 6, 8, and 9). Different letters indicate statistically significant differences (*p* < 0.05).

Color differences between control and probiotic-loaded capsules are shown in Figure 4. The most pronounced color changes following the incorporation of *L. rhamnosus* and *B. clausii* were observed in formulations with the highest probiotic loading, likely reflecting increased matrix density and compositional complexity. When ΔE values exceeded 2, color differences were perceptible to the naked eye, as observed particularly between samples 1–3 and 7–9. Overall, increasing ST and probiotic content resulted in greater color variation, with the most evident changes occurring in samples 1–3. These findings indicate that both polymer ratio and probiotic concentration significantly influence capsule appearance, which may be relevant for consumer perception and functional quality.

### 2.2. Encapsulation Efficiency and Viability of Encapsulated Probiotics

**Figure 5:**
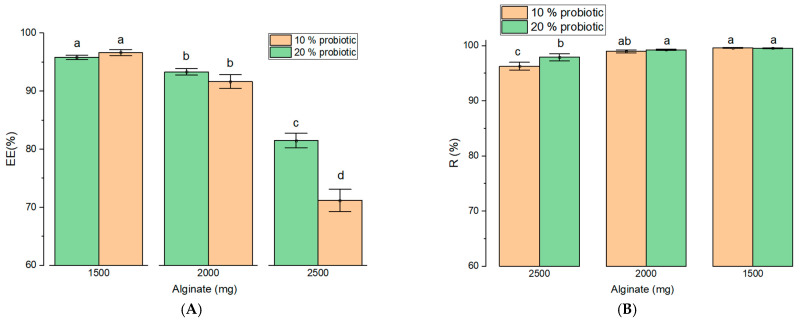

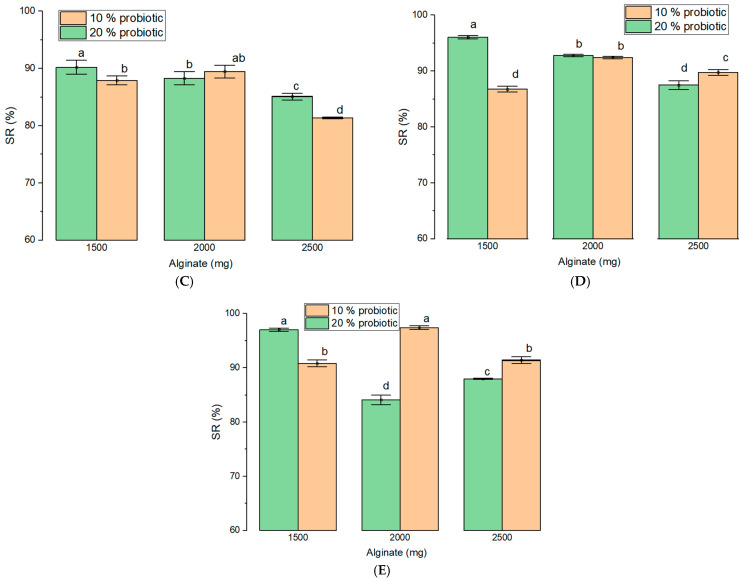
Influence of formulation variables on probiotic encapsulation and survival (SR). (**A**) Encapsulation efficiency (EE) of capsule formulations; (**B**) probiotic viability (R) following encapsulation; (**C**) survival after 1 h exposure to simulated gastric fluid; (**D**) survival after 1 h exposure to simulated intestinal fluid; and (**E**) cumulative survival following sequential exposure to simulated gastric (1 h) and intestinal (1 h) fluids. Data are presented as mean ± SEM. Different letters indicate statistically significant differences (*p* < 0.05).

Encapsulation efficiency (EE) ranged from 71.20% to 96.67% and was strongly influenced by both matrix composition and probiotic loading (Figure 5A).

EE increased as SA decreased within the tested SA-ST combinations. Thus, the highest EE was achieved at the lowest SA level (1500 mg), likely due to the space-filling properties of ST, which enhance matrix density and probiotic entrapment. Probiotic loading also exerted a significant effect (*p* < 0.001), with higher initial loads (20%) generally resulting in improved EE, particularly at higher SA concentrations, as reflected by a significant interaction effect (*p* < 0.001). Thus, the lowest EE was observed for sample 2 (10% probiotic; 71.20 ± 1.09%). In contrast, formulations with increased probiotic content or a more balanced SA-ST ratio exhibited higher EE values, including sample 3 (20% probiotic; 81.50 ± 0.72%), sample 5 (20% probiotic; 91.67 ± 0.69%), and sample 6 (10% probiotic; 93.33 ± 0.32%), the latter containing a higher ST proportion than samples 2, 3, and 5. The highest encapsulation efficiencies were achieved in samples with equal SA and ST content, namely sample 8 (10% probiotic; 96.67 ± 0.30%) and sample 9 (20% probiotic; 95.83 ± 0.23%). These findings indicate that optimal probiotic retention is achieved through a favorable combination of SA-ST matrix composition and probiotic loading.

Overall, the enhancement in EE was most pronounced in ST-rich formulations, indicating that ST incorporation plays a key role in improving encapsulation performance. Notably, the encapsulation efficiencies achieved in ST-containing formulations exceeded those previously reported for SA systems incorporating prebiotics such as fructooligosaccharides (FOS), for which a maximum EE of 91% was reported at 50% FOS inclusion [24]. Cell viability following encapsulation remained consistently high across all formulations (>96%; Figure 5B). Statistical analysis revealed that polymer ratio significantly influenced viability (*p* < 0.001), with slightly higher yields observed as ST content increased, whereas probiotic concentration had no significant effect on cell viability (*p* > 0.05). The present results indicate that the proposed extrusion method favored probiotic encapsulation efficiency (Figure 5E) compared with layer-by-layer approaches. This enhanced performance may be attributed to the smooth capsule surface produced by extrusion, which enables SA and ST to function more effectively as carrier matrices for *L. rhamnosus* and *B. clausii* than systems based on chitosan or casein [25].

Probiotic survival was evaluated sequentially in simulated gastric fluid (SGF) and simulated intestinal fluid (SIF). In SGF, survival rates ranged from 81.38% to 90.24% (Figure 5C), with both polymer ratio and probiotic load exerting significant effects (*p* < 0.001). Formulations with lower SA and higher ST content provided enhanced protection under acidic conditions. In SIF, survival patterns were more complex and dominated by a strong interaction between formulation variables (*p* < 0.001; Figure 5D). For formulations containing 10% probiotics, survival increased with SA content up to 2000 mg and then declined, whereas at a 20% probiotic load, maximal survival was observed at the lowest SA level (1500 mg). Cumulative survival following sequential exposure to SGF and SIF was highest (97.05%) for capsules containing equal amounts of SA and ST (1500:1500) with 20% probiotic loading. These results define an optimal formulation window, in which 10% probiotic formulations performed best at intermediate polymer ratios (2000:1000), while 20% formulations benefited most from ST-rich matrices (1500:1500). Consistent with previous reports, ST-based systems enhance probiotic stability during gastrointestinal transit [7]. In addition to improving protection of higher biomass loads, ST incorporation supports controlled matrix solubilization and contributes to a cost-effective manufacturing process.

The combined use of SA and ST also improved resistance to capsule degradation under intestinal conditions. Whereas SA-only capsules have been reported to undergo complete bacterial release after approximately 40 min in simulated intestinal fluids [26], ST incorporation significantly enhanced structural integrity, allowing capsules to maintain uniform shape without network disruption, even at high SA content. In contrast, hydrogel capsules prepared from gelatin and transglutaminase exhibit irregular structures with large pores and compromised network integrity, limiting their effectiveness as probiotic carriers [27]. Similar limitations have been reported for SA–pectin systems, which display increased porosity and surface irregularities [28], as well as for SA–cellulose formulations, which require cryoprotectants to preserve capsule integrity [29]. Notably, the SA-ST system developed in this study did not require cryoprotectants, an important advantage given that such additives can adversely affect capsule rheology, structural stability, and long-term probiotic viability.

### 2.3. Swelling Behavior and Kinetics of Capsules

All formulations exhibited a progressive increase in swelling ratio (S) over the 24 h period (Table 1), with distinct differences attributable to matrix composition and probiotic loading.

At 1 h, S values ranged from 1.95% (sample 4) to 19.60% (sample 2), reflecting formulation-dependent differences in early water uptake. During the first 8 h, swelling increased markedly in all samples, with ST-rich formulations containing probiotics (samples 8 and 9) reaching higher S values (32.08% and 36.90%, respectively), indicative of a more flexible matrix and enhanced hydration capacity. In contrast, SA-rich control formulations (sample 1) exhibited more moderate swelling (16.38%), consistent with a denser polymer network and more restricted expansion.

Swelling continued to increase at 12 and 24 h, with the highest S observed in formulations containing equal SA-ST ratios and higher probiotic loading, namely sample 8 (50.11%) and sample 9 (57.85%). These findings demonstrate that both probiotic incorporation and polymer composition directly influence the water absorption behavior of the capsules. Analysis of swelling kinetics (Table 1) revealed pronounced matrix-dependent effects. Formulations with lower SA content (1500 mg) displayed the fastest and most extensive swelling, particularly when loaded with probiotics, in agreement with previous reports showing enhanced swelling of SA beads upon incorporation of additional polysaccharides [30,31]. The inclusion of ST in biopolymeric hydrogels is known to increase swelling capacity by promoting matrix hydration [32]. Notably, the formulation containing 2500 mg SA and 10% probiotic exhibited increased variability at later time points, suggesting heterogeneity in matrix structure. In ST-rich matrices (1500 mg SA), probiotic addition acted as a swelling promoter, increasing mean S from approximately 37% (0% probiotic) to nearly 58% (20% probiotic), likely due to increased matrix porosity or hydrophilicity. Conversely, in SA-rich matrices (2000–2500 mg), probiotic incorporation acted as a swelling restrictor; for example, in the 2000 mg SA formulation, S decreased from ~56% to ~40% upon addition of 20% probiotics, likely reflecting physical obstruction of polymer chain relaxation or occupation of free volume by bacterial cells.

The area under the curve (AUC) analysis reveals a clear dependence of cumulative swelling behavior on both polymer composition and probiotic loading. For all SA:ST ratios, the incorporation of probiotics leads to an increase in AUC, indicating enhanced overall swelling capacity compared with control capsules. This effect is more pronounced at higher probiotic concentrations (20%), suggesting that probiotic incorporation promotes matrix hydration and water uptake over time by creating additional diffusion pathways or by modifying the polymer network structure. This effect was more pronounced in formulations with lower SA content (samples 4–9), where increased ST and probiotic levels promoted matrix solubilization. The observed increase in S can be attributed to structural alterations of the gel network induced by encapsulated cells.

In addition to swelling kinetics in PBS, capsule behavior was further evaluated under simulated gastrointestinal conditions to assess pH-responsive hydration. According to the results, all formulations showed behavior suitable for targeted intestinal delivery. In SGF, swelling remained limited across all compositions, even at high probiotic loadings (6.0–23.2%). Control capsules exhibited swelling indices of 6.0–9.1%, increasing with ST content: the lowest swelling was observed for the 2500:500 SA-ST ratio (~6.0%), while the 2000:1000 and 1500:1500 ratios reached approximately 8.2% and 9.1%, respectively.

Probiotic incorporation led to a progressive increase in swelling for all formulations. For the 1500:1500 ratio, swelling increased from 9.1% (0% probiotics) to 16.2% (10%) and 23.2% (20%), whereas formulations with higher SA content showed lower absolute values, remaining below 15% even at 20% probiotic loading for the 2500:500 ratio. The 2000:1000 formulation displayed intermediate behavior, reflecting a balance between SA-driven structural stability and the hydrophilic contribution of ST.

Limited swelling in SGF is attributed to the acidic environment, which induces contraction of the SA matrix and restricts water uptake, thereby preserving capsule integrity and protecting encapsulated probiotics [33]. In contrast, swelling increased markedly in SIF, ranging from 6.5% to 59.8%. For control formulations, swelling reached 6.6%, 12.3%, and 18.1% with increasing ST content, reflecting a less compact and more hydrophilic polymer network. The higher pH of SIF promotes ion exchange and relaxation of the SA-ST matrix, enabling greater water penetration and capsule expansion.

Formulations with higher ST content (1500 mg) and increased probiotic loading exhibited the greatest swelling, reaching 59.8% for the 1500:1500 SA-ST ratio with 20% probiotics. Probiotic incorporation enhanced swelling in all formulations, with a stronger effect under intestinal conditions. For example, swelling increased from 18.1% (0% probiotics) to 42.8% (10%) and 59.8% (20%) for the 1500:1500 formulation. In the combined SGF + SIF test, swelling indices were intermediate, reflecting the precontracted SA network formed in acidic conditions that limits subsequent expansion (e.g., 23.2% in SGF, 59.8% in SIF, and 41.98% in SGF + SIF for the 1500:1500 formulation with 20% probiotics).

Mechanical interactions and electrostatic repulsion between negatively charged bacterial surfaces and free SA chain segments may weaken the polymer network, thereby enhancing water retention [34]. Consistent with previous reports, probiotic incorporation can either enhance or suppress swelling depending on the polymer system and formulation context. For example, the inclusion of bacterial cells in pectin-based films resulted in a slight decrease in swelling, attributed to reduced hydrophilic content within the matrix [35]. Similarly, reduced swelling has been reported for SA capsules containing *L. rhamnosus* in the presence of calcium chloride and disodium hydrogen phosphate, indicating that probiotic incorporation alone may restrict matrix expansion in SA-dominant systems [36].

Overall, the swelling response demonstrates high gastric stability and pronounced expansion under intestinal conditions, supporting controlled and targeted probiotic release. Similar trends have been reported for SA-based systems delivering Lactobacillus species for intestinal targeting [37]. Compared with ST–chitosan matrices, the SA-ST formulations evaluated here exhibit more controlled swelling, highlighting the importance of polymer composition and matrix structure, rather than capsule size alone, in regulating probiotic release and survival [38,39]. Importantly, swelling characteristics were governed by the combined effect of SA, ST, and probiotic content. Lower SA (higher ST) formulations promoted increased swelling in the presence of probiotics, which may favor rapid release applications. In contrast, higher SA concentrations limited matrix expansion upon probiotic incorporation, supporting sustained release and enhanced protection in the upper gastrointestinal tract.

## 3. Conclusions 

This study developed biopolymer-based capsules composed of SA and wheat ST for the efficient encapsulation of *L. rhamnosus* and *B. clausii*, achieving encapsulation efficiencies ranging from 71.20% to 96.67% and enabling controlled release. The SA-ST combination yielded relatively small, compact capsules (236.6–279.17 μm) that effectively protected the probiotics under simulated gastric conditions while promoting controlled release in simulated intestinal fluids. The results indicated that the formulation with SA and ST in a 1:1 ratio can be used as a matrix for the incorporation of *L. rhamnosus* and *B. clausii*, providing targeted and sustained release, and showing the highest encapsulation efficiency and cell viability rate. Formulations with a higher SA content are useful for prolonged stability in high-moisture environments, such as certain food products like yogurt, due to their lower swelling ratio and enhanced structural stability. Although probiotic and ST incorporation increased the swelling ratio, capsule hydration in aqueous media remained below 57.86%, indicating favorable structural stability and functional adaptability of the polymer matrix. In vitro evaluations confirmed capsule integrity and sustained probiotic viability throughout gastrointestinal simulation. Future studies should focus on tolerance to bile salts and the inclusion of digestive enzymes in more physiologically relevant gastrointestinal models to better simulate dynamic in vivo conditions. The effect of different temperatures and sodium chloride concentrations on cell viability should also be investigated, together with long-term stability tests under various storage conditions to evaluate industrial applicability, including the assessment of capsule integrity and rehydration behavior following controlled drying processes. Furthermore, the cytocompatibility of the capsules, with varying biopolymer-to-probiotic ratios, should assessed through direct and indirect viability tests on human epithelial cell lines to support potential in vivo applications. The incorporation of these capsules into food matrices will allow evaluation of probiotic behavior under realistic gastrointestinal environments, while in vivo investigations will be necessary to validate their effects on gut microbiota composition, functionality, and host health outcomes.

## 4. Materials and Methods

### 4.1. Materials

SA from brown algae (medium viscosity, ≥2000 cP at 2% at 25 °C), unmodified wheat ST (≤0.3% protein content), anhydrous calcium chloride (94% assay), simulated gastric fluid without enzymes (pH 1.1–1.3 at 25 °C, prepared by diluting the commercial stock solution to a final volume of 6 L with distilled water, according to the manufacturer’s instructions), phosphate-buffered saline (PBS; pH 7.2–7.6, prepared according to the manufacturer’s specifications and verified before each experiment), and analytical-grade distilled water were obtained from Sigma-Aldrich Romania.

*L. rhamnosus* ATCC 7469 (American Type Culture Collection, Manassas, VA, USA, distributed by Mediclim, Otopeni, Romania) and *B. clausii* (SunWave Pharma, Romanian Distribution Branch, Bucharest, Romania) were reactivated twice on MRS agar at 24 °C for 24 h. Following the second reactivation, a 16 h culture was used for encapsulation in SA-ST matrices. Capsules were prepared using the extrusion method as previously described [40]. All utensils, glassware, and distilled water were sterilized by autoclaving at 121 °C for 20 min. Capsule formulations with varying SA and ST concentrations (*w*/*v*), containing *L. rhamnosus* and *B. clausii*, were prepared as summarized in Table 2.

### 4.2. Methods

#### 4.2.1. Capsule Development

**Figure 6:**
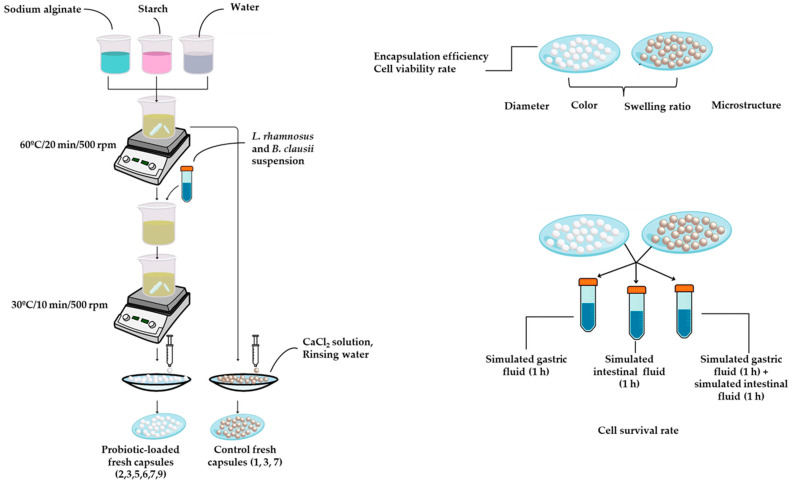
Workflow diagram of the biopolymer-based gel capsule preparation process.

The biopolymer mixture was homogenized in 150 mL of distilled water under continuous stirring (500 rpm) and maintained at 60 °C for 20 min to ensure complete solubilization and uniform mixing. In parallel, separate suspensions of *L. rhamnosus* and *B. clausii* were prepared and adjusted to a McFarland standard of 5.24, corresponding to approximately 1.57 × 10^9^ CFU/mL. The two suspensions were then combined in equal volumes to obtain a mixed probiotic suspension, ensuring equivalent contributions from each strain. The mixed probiotic suspension was added to the capsule-forming solution at 10% or 20% (*v*/*v*) and homogenized at 500 rpm for 10 min at 30 °C. The resulting mixture was extruded dropwise into a 5% (*w*/*v*) CaCl_2_ solution and maintained for 10 min to allow capsule shell formation. The capsules were subsequently rinsed with distilled water and stored under refrigeration until further analysis (Figure 6).

#### 4.2.2. Physical and Optical Characterization of Capsules

Capsule diameter, color parameters, swelling ratio, encapsulation efficiency, and behavior in simulated gastric and intestinal fluids were evaluated. Capsule diameter was measured using a digital Yato micrometer (Shanghai, China) with a resolution of 0.001 mm. Ten individual fresh capsules randomly selected from each formulation and gently removed from the CaCl_2_ solution, blotted to remove excess surface moisture, and measured separately without compression.

Capsule surface morphology was examined using a scanning electron microscope (Tescan Vega 3 LMH, Tescan Orsay Holding, Brno, Czech Republic) equipped with an EDS detector (Oxford Instruments, Abingdon, UK), operating at an accelerating voltage of 20 kV. SEM analysis was used to assess capsule morphology and encapsulation efficiency. Fresh capsules were fractured to expose internal structures for evaluation. Chemical composition and potential interactions between capsule components were analyzed by Fourier Transform Infrared (FTIR) spectroscopy. Capsule contents were crushed, mixed with KBr, and pressed into pellets prior to analysis. Infrared spectra were recorded in the 1000–4000 cm^−1^ range, corresponding to characteristic molecular bond vibrations.

Capsule color was assessed using a Konica Minolta CR-400 colorimeter, calibrated with a standard white plate (L* = 95.46, a* = −0.56, b* = −4). Color parameters L*, a*, and b* were recorded in the CIELab color space for each formulation, with ten replicates performed to ensure measurement accuracy and reproducibility. Color differences (ΔE) were calculated according to the following equation:

(1) $$\Delta E=\sqrt{\left\{{\left(L_{2}^{*}-L_{1}^{*}\right)}^{2}+{\left(a_{2}^{*}-a_{1}^{*}\right)}^{2}+{\left(b_{2}^{*}-b_{1}^{*}\right)}^{2}\right\}}$$

where L* represents luminosity (0 = black, 100 = white), a* is the position on the green–red axis (negative values → green, positive → red), and b*, the position on the blue–yellow axis (negative values → blue, positive → yellow). The symbol 2 refers to the capsules containing probiotics (samples 2, 3, 5, 6, 8 and 9), while 1 represents the control samples, without probiotics (samples 1, 4 and 7). The use of instrumental CIELab analysis ensures objective and reproducible assessment of color differences, minimizing potential bias associated with subjective visual evaluation.

#### 4.2.3. Encapsulation Efficiency and Viability of Encapsulated Probiotics

Encapsulation efficiency (EE) was determined exclusively for probiotic-containing formulations (samples 2, 3, 5, 6, 8 and 9). Viable encapsulated cells were quantified by dissolving 0.2 g of freshly prepared gel capsules in 9.8 mL of sterile sodium citrate solution (1%, pH 6) and gently homogenizing the mixture at room temperature for approximately 15 min. Serial dilutions (1:100) were then prepared, and 1 mL aliquots were plated on MRS agar. Plates were incubated under anaerobic conditions at 37 °C for 72 h. Encapsulation efficiency was calculated using the following equation:

(2) $$EE,\%=\frac{N_{1}}{N_{0}}\times 100$$

where $N_{1}$ represents the number of viable cells recovered from the gel capsules and $N_{0}$ denotes the number of viable cells in the initial *L. rhamnosus* and *B. clausii* emulsion. Results are expressed as CFU/mL.

The viability rate of encapsulated cells (*R*, %) following the microencapsulation process was calculated using Equation (3), as previously described [41]:

(3) $$R,\;\%=\;\frac{{log}_{10}N_{0}}{{log}_{10}N_{1}}\;\times \;100$$

where ${log}_{10}N_{0}$ represents the number of viable cells encapsulated within the capsules and ${log}_{10}N_{1}$ denotes the number of free viable cells initially added to the emulsion. Results are expressed as CFU/mL.

Evaluation of capsule behavior under gastrointestinal conditions was performed using simulated gastric and simulated intestinal fluids to mimic in vitro physiological environments. The simulated fluids were prepared according to the manufacturer’s instructions. For each test, 0.2 g of fresh gel capsules were immersed in 9.8 mL of the corresponding solution, following the experimental workflow shown in Figure 6.

To evaluate probiotic survival under simulated gastric and intestinal conditions, viable cells encapsulated within the matrix were quantified as follows. Fresh capsules (0.2 g) were immersed in 9.8 mL of simulated gastric fluid or simulated intestinal fluid, depending on the condition tested. For sequential gastrointestinal simulation, capsules were first exposed to simulated gastric fluid for 1 h, followed by transfer to simulated intestinal fluid for an additional 1 h. During each exposure, samples were gently shaken at room temperature for 10–12 min to facilitate bacterial release from the capsules. Following release, serial dilutions were prepared in triplicate using sterile distilled water up to a final dilution of 1:100. Aliquots (1 mL) from the final dilution were plated in triplicate onto MRS agar plates and incubated under anaerobic conditions at 37 °C for 72 h. Viable cell counts were expressed as CFU/g of capsules.

#### 4.2.4. Swelling Behavior and Kinetics of Capsules

For swelling ratio (S) determination, 100 mg of fresh capsules from each formulation were immersed in phosphate-buffered saline (PBS). At predetermined time points (1, 2, 3, 4, 5, 6, 8, 12 and 24 h), capsules were removed, gently blotted with filter paper to remove excess surface liquid, and reweighed. The swelling ratio was calculated using the following equation:

(4) $$S\;(\%)=\frac{W_{1}-W_{0}}{W_{0}}\times 100\;$$

where $W_{1}$ represents the capsule mass after swelling and $W_{0}$ denotes the initial mass of the fresh capsule.

The swelling index was measured for samples in simulated gastric and intestinal fluids. Five fresh capsules were randomly selected, weighed, and then immersed in gastric fluid for one-hour, intestinal fluid for one hour, and in a combination of gastric and intestinal fluids (one hour each). Swelling kinetics were further assessed by calculating the area under the swelling ratio–time curve (AUC), which reflects the rate and extent of swelling as a function of matrix density, according to the equation:

(5) $$\mathrm{AUC}=\int_{0}^{24h}S(t)\;dt$$

A higher AUC value indicates faster and more extensive capsule swelling, reflecting greater water absorption capacity and a less dense polymer matrix. Conversely, lower AUC values correspond to a more rigid network with reduced swelling over time. The parameter T_50_ represents the time required for the swelling ratio to reach 50% of its maximum value during the 24 h testing period. Together, AUC and T_50_ provide complementary measures of swelling extent and kinetics, with lower T_50_ values indicating more rapid hydration of the polymer matrix. Unless otherwise stated, all experiments were performed in three independent replicates for each formulation. Capsule diameter and color measurements were conducted using 10 independent capsules per formulation.

Statistical Analysis: Primary analyses were conducted using ordinary least squares (OLS) models with categorical main effects. Two-way analysis of variance (ANOVA) was applied to assess the effects of formulation composition and probiotic load, as well as their interaction. For time-dependent swelling data, a global OLS model including time × independent variable interactions was fitted. Pairwise comparisons were performed using Tukey’s Honest Significant Difference (HSD) test within each factor, interaction, or time point. Data are presented as mean ± SEM, and statistical significance was set at *p* < 0.05. All analyses were performed using OriginPro 2024 (64-bit) SR1 (version 10.1.0.178, Academic; OriginLab Corporation, Northampton, MA, USA).

## Figures and Tables

**Table 1:** Swelling ratio (S, %) of SA/ST probiotic-loaded capsules at different immersion times (1–24 h).

Sample *	Time (h)								
	1	2	3	4	5	6	8	12	24
1	4.8 ± 0.005 ^a^	6.4 ± 0.011 ^b^	8.2 ± 0.017 ^c^	10.3 ± 0.023 ^d^	13.0 ± 0.017 ^e^	13.5 ± 0.011 ^f^	16.4 ± 0.057 ^g^	17.6 ± 0.005 ^h^	20.4 ± 0.017 ^i^
2	19.6 ± 0.011 ^a^	19.7 ± 0.017 ^a^	20.1 ± 0.023 ^b^	23.2 ± 0.005 ^c^	25.6 ± 0.051 ^d^	26.3 ± 0.288 ^d^	28.8 ± 0.577 ^d^	34.8 ± 0.023 ^e^	44.2 ± 0.871 ^f^
3	3.2 ± 0.063 ^a^	7.2 ± 0.127 ^b^	10.8 ± 0.115 ^c^	18.4 ± 0.231 ^d^	24.6 ± 1.18 ^d^	27.4 ± 0.196 ^e^	28.3 ± 0.086 ^e^	29.8 ± 0.052 ^f^	33.3 ± 1.714 ^ef^
4	1.9 ± 0.144 ^a^	10.8 ± 0.005 ^b^	11.2 ± 0.069 ^b^	16.4 ± 0.692 ^c^	21.1 ± 0.583 ^c^	23.3 ± 0.583 ^c^	28.5 ± 1.166 ^d^	32.5 ± 0.635 ^d^	56.5 ± 0.346 ^e^
5	11.8 ± 0.635 ^a^	12.8 ± 0.132 ^a^	15.2 ± 0.577 ^a^	21.4 ± 0.658 ^b^	23.1 ± 0.046 ^b^	24.2 ± 1.212 ^b^	32.9 ± 0.594 ^c^	38.3 ± 0.594 ^c^	23.6 ± 0.190 ^b^
6	11.1 ± 0.59 ^a^	15.0 ± 0.028 ^a^	17.2 ± 1.154 ^a^	21.6 ± 0.594 ^b^	22.2 ± 0.346 ^b^	28.9 ± 1.166 ^b^	30.8 ± 0.692 ^c^	38.5 ± 1.137 ^c^	39.5 ± 0.588 ^d^
7	3.3 ± 0.015 ^a^	11.5 ± 0.288 ^b^	20.4 ± 0.115 ^c^	25.1 ± 2.309 ^bc^	28.6 ± 0.692 ^d^	29.8 ± 1.460 ^cd^	31.8 ± 0.600 ^d^	34.7 ± 0.635 ^d^	37.3 ± 0.683 ^e^
8	12.9 ± 1.270 ^a^	15.4 ± 0.069 ^a^	18.4 ± 0.739 ^a^	20.6 ± 0.560 ^b^	22.7 ± 0.525 ^b^	24.4 ± 0.692 ^b^	32.0 ± 0.583 ^c^	40.9 ± 0.231 ^d^	50.1 ± 0.583 ^e^
9	13.4 ± 0.606 ^f^	15.9 ± 0.461 ^c^	16.8 ± 0.462 ^e^	21.5 ± 0.883 ^a^	23.6 ± 1.218 ^f^	27.9 ± 0.588 ^d^	36.9 ± 0.259 ^de^	40.8 ± 0.323 ^b^	57.8 ± 0.635 ^a^

* 1–9 represent sample codes. Values are expressed as mean ± SEM. Means followed by different letters indicate statistically significant differences (*p* ≤ 0.05).

**Table 2:** Capsule formulations with varying SA and ST proportions.

Code	SA, mg	ST, mg	*L. rhamnosus* and *B. clausii*, %
1	2500	500	-
2	2500	500	10
3	2500	500	20
4	2000	1000	-
5	2000	1000	10
6	2000	1000	20
7	1500	1500	-
8	1500	1500	10
9	1500	1500	20

SA—sodium alginate, ST—starch.

## Data Availability

The original contributions presented in this study are included in the article. Further inquiries can be directed to the corresponding author.

## Abbreviations

The following abbreviations are used in this manuscript:

AUC	Area Under Curve
CFU	Colony Forming Unit
EE	Encapsulation Efficiency
FDA	US Food and Drug Administration
FOS	Fructooligosaccharides
HSD	Honest Significant Difference
OLS	Ordinary Least Squares
MRS agar	de Man, Rogosa and Sharpe agar
PBS	Phosphate-Buffered Saline
R	Viability Rate
S	Swelling Ratio
SA	Sodium Alginate
SEM	Standard Error of the Mean
SR	Survival Rate
ST	Starch
